# Circadian pathway genetic variation and cancer risk: evidence from genome-wide association studies

**DOI:** 10.1186/s12916-018-1010-1

**Published:** 2018-02-19

**Authors:** Simone Mocellin, Saveria Tropea, Clara Benna, Carlo Riccardo Rossi

**Affiliations:** 1 Department of Surgery Oncology and Gastroenterology, University of Padova, Via Giustiniani 2, 35128 Padova, Italy; 2 Istituto Oncologico Veneto, IOV-IRCCS, Padova, Italy

**Keywords:** Circadian clock, Gene pathway, Genetic variation, Single nucleotide polymorphism (SNP), Germline, Cancer risk, Cancer predisposition, Genome-wide association study (GWAS), Pathway analysis

## Abstract

**Background:**

Dysfunction of the circadian clock and single polymorphisms of some circadian genes have been linked to cancer susceptibility, although data are scarce and findings inconsistent. We aimed to investigate the association between circadian pathway genetic variation and risk of developing common cancers based on the findings of genome-wide association studies (GWASs).

**Methods:**

Single nucleotide polymorphisms (SNPs) of 17 circadian genes reported by three GWAS meta-analyses dedicated to breast (Discovery, Biology, and Risk of Inherited Variants in Breast Cancer (DRIVE) Consortium; cases, *n* = 15,748; controls, *n* = 18,084), prostate (Elucidating Loci Involved in Prostate Cancer Susceptibility (ELLIPSE) Consortium; cases, *n* = 14,160; controls, *n* = 12,724) and lung carcinoma (Transdisciplinary Research In Cancer of the Lung (TRICL) Consortium; cases, *n* = 12,160; controls, *n* = 16,838) in patients of European ancestry were utilized to perform pathway analysis by means of the adaptive rank truncated product (ARTP) method. Data were also available for the following subgroups: estrogen receptor negative breast cancer, aggressive prostate cancer, squamous lung carcinoma and lung adenocarcinoma.

**Results:**

We found a highly significant statistical association between circadian pathway genetic variation and the risk of breast (pathway *P* value = 1.9 × 10^–6^; top gene RORA, gene *P* value = 0.0003), prostate (pathway *P* value = 4.1 × 10^–6^; top gene ARNTL, gene *P* value = 0.0002) and lung cancer (pathway *P* value = 6.9 × 10^–7^; top gene RORA, gene *P* value = 2.0 × 10^–6^), as well as all their subgroups. Out of 17 genes investigated, 15 were found to be significantly associated with the risk of cancer: four genes were shared by all three malignancies (*ARNTL*, *CLOCK*, *RORA* and *RORB*), two by breast and lung cancer (*CRY1* and *CRY2*) and three by prostate and lung cancer (*NPAS2*, *NR1D1* and *PER3*), whereas four genes were specific for lung cancer (*ARNTL2*, *CSNK1E*, *NR1D2* and *PER2*) and two for breast cancer (*PER1*, *RORC*).

**Conclusions:**

Our findings, based on the largest series ever utilized for ARTP-based gene and pathway analysis, support the hypothesis that circadian pathway genetic variation is involved in cancer predisposition.

**Electronic supplementary material:**

The online version of this article (10.1186/s12916-018-1010-1) contains supplementary material, which is available to authorized users.

## Background

The circadian clock is a time-tracking rhythmic biological system (internal timing machine) with a periodicity of about 24 h that enables organisms to anticipate environmental changes (such as food availability) and allows them to modify their behaviour and physiological functions (e.g. sleep and wakefulness, basal metabolism, body temperature, blood pressure, hormone production and immunity) in the most efficient way [1]. This system consists of two components: the central clock, located in the suprachiasmatic nucleus of the brain, and the peripheral clocks, which are present in virtually all body tissues. Circadian rhythms are controlled by what are called circadian pathway genes [2], which have been discovered in all studied species: remarkably, the disruption of these rhythms has been linked to the risk of different diseases such as insomnia, depression, jet leg, stomach ailments, heart attack and cancer [3]. As regards the latter, a growing wealth of evidence supports the potential tumour suppressor role of the biological clock [4]. In particular, single germline variations of circadian genes have been associated with the predisposition of some tumour types such as breast carcinoma [5], although the evidence is not conclusive due to the scarcity of data in this recent field of research.

Germline DNA variation has been long recognized as a key component of the individual risk to develop cancer, and recently the discovery rate of susceptibility loci is being greatly accelerated by genome-wide association studies (GWASs) which can test up to one million single nucleotide polymorphisms (SNPs) in thousands of subjects at a time [6]. However, the proportion of genetic susceptibility to complex traits (such as cancer) explained by single locus analysis still remains small, whereas it is increasingly recognized that multiple locus analysis — such as gene and gene set (or pathway) analysis — is more powerful for dissecting the genetic architecture of complex diseases according to the principles of systems genetics [7]. In fact, a single SNP can have an effect too small to be detected by the single locus approach, whereas gene/pathway analysis, which jointly tests multiple SNPs from the same gene/pathway, can more likely identify the association between the outcome and the basic functional unit involved in disease development [8–10].

In this work, we intended to investigate whether germline genetic variation of the circadian pathway is associated with the risk of cancer by analysing publicly available data from GWASs. To this aim we chose to focus on the three most common tumour types, i.e. lung, breast and prostate carcinomas, which account for up to 40% of all cancer incident cases and observed deaths [11].

## Methods

### Study design

We conducted this study to test the hypothesis that germline DNA variation of the circadian pathway might be associated to the risk of cancer. To this aim we followed the principles described in the Strengthening the Reporting of Genetic Association Studies (STREGA) statement, an extension of the Strengthening the Reporting of Observational Studies in Epidemiology (STROBE) statement (www.strobe-statement.org) [12].

Briefly, the study was composed of three phases: (1) identification of circadian genes; (2) collection of single nucleotide variants of these genes that have been associated with the risk of the three most common malignancies; (3) conduction of adaptive rank truncated product (ARTP)-based gene and pathway analysis based on the *P* values of circadian gene SNPs retrieved from GWASs.

To this aim, we first defined the core circadian genes by querying the publicly available Molecular Signatures Database (MSigDB), which includes compiled gene sets from a variety of resources, such as the Kyoto Encyclopedia of Genes and Genomes (KEGG, www.genome.jp/kegg), Gene Ontology (GO, www.geneontology.org) and others [13]. We also screened previously published literature dedicated to the circadian clock [1, 2].

**Fig. 1:**
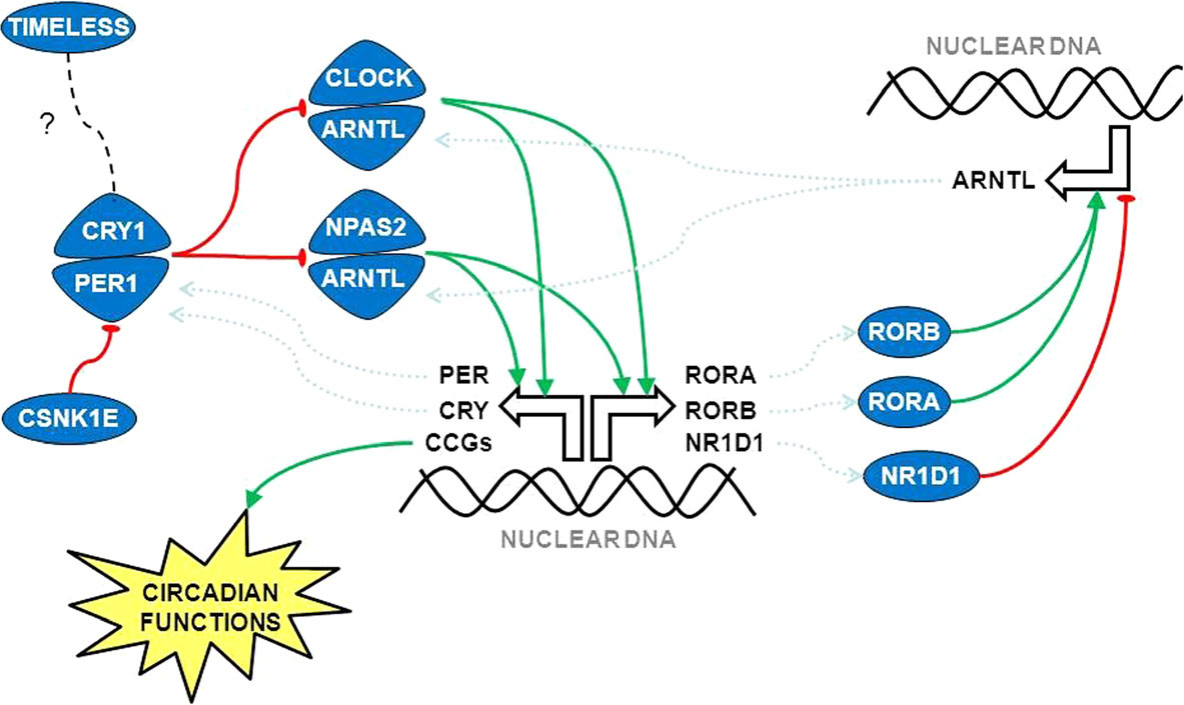
Schematic view of the circadian pathway. CLOCK and NPAS2 form heterodimers with ARNTL (also known as BMAL1) or ARNTL2 (BMAL2); these heterodimers act as transcription factors binding to enhancer box (E-box) elements upstream of target genes. Besides the clock-controlled genes (CCGs), which mediate the circadian pathway physiological functions, *CLOCK* and *NPAS2* activate the transcription of other core circadian genes such as *PER1*, *PER2*, *PER3* and *CRY1*, *CRY2*. PER and CRY proteins heterodimerize and activate a negative feedback loop acting directly on CLOCK and NPAS2. The activity of PER and CRY proteins is also regulated by additional proteins such as CSNK1E and CSNK1D (inhibition) and TIMELESS (unclear effect), respectively. CLOCK and NPAS2 also transactivate the expression of other pathway components such as NR1D1, NR1D2 (also known as REV-ERBs) and RORA, RORB and RORC (which are transcription factors acting through ROR/REV-ERB elements): these proteins can inhibit or enhance ARNTL transcription, respectively, which adds a further level of modulation of CLOCK/NPAS2 activity. *Green lines*, stimulatory effect (positive loop); *red lines*, inhibitory effect (negative loop)

We identified and studied the following 19 genes: *ARNTL* (*aryl hydrocarbon receptor nuclear translocator-like*), *ARNTL2* (*aryl hydrocarbon receptor nuclear translocator-like 2*), *CLOCK* (*clock circadian regulator*), *CRY1* (*cryptochrome circadian clock 1*), *CRY2* (*cryptochrome circadian clock 2*), *CSNK1D* (*casein kinase 1 delta*), *CSNK1E* (*casein kinase 1 epsilon*), *NPAS2* (*neuronal PAS domain protein 2*), *NR1D1* (*nuclear receptor subfamily 1 group D member 1*), *NR1D2* (*nuclear receptor subfamily 1 group D member 2*), *PER1* (*period circadian clock 1*), *PER2* (*period circadian clock 2*), *PER3* (*period circadian clock 3*), *RORA* (*RAR-related orphan receptor A*), *RORB* (*RAR-related orphan receptor B*), *RORC* (*RAR-related orphan receptor C*), *TIMELESS* (*timeless circadian clock*), *BHLHE40* (*basic helix-loop-helix family member E40*), *BHLHE41* (*basic helix-loop-helix family member E41*). The functional interactions of their protein products are illustrated in Fig. 1.

The physical position of these genes (including 3000 bp upstream and 1000 bp downstream) — which was needed to retrieve the relevant SNPs — was assessed using the National Center for Biotechnology Information (NCBI) Gene database (https://www.ncbi.nlm.nih.gov/gene).

Then we searched the NCBI database of genotypes and phenotypes (GaPdb) data repository (https://www.ncbi.nlm.nih.gov/gap) as the source of publicly available GWAS findings on the three most frequently occurring tumour types: breast, prostate and lung carcinomas.

To be eligible, the data had to be from a GWAS and include the following information: (1) variant ID of the SNP, which allows one to know the variant physical position (with special regard to the relationship with the gene of interest) as well as its effect and reference alleles; (2) strength of association as expressed by the odds ratio (OR); (3) *P* value of the association test.

GWAS meta-analyses were deemed to be more informative than single GWASs due to the larger sample sizes achieved by consortia pooling the findings of multiple GWASs.

### ARTP-based gene and pathway analysis

Gene and pathway analysis was carried out using the ARTP method — which was originally designed to analyse individual-level genotype data — extended to accept input from SNP-level summary statistics (summary-based ARTP, sARTP), as performed by the ARTP2 (version 0.9.22) R package [14, 15].

Briefly, the ARTP method was developed to overcome the major limitations of other existing *P*-value-combining approaches (such as the Fisher’s product method and the rank truncated product), which do not take into consideration the organization of the DNA into functional elements (that is, genes), ignore the linkage disequilibrium patterns between SNPs within the same gene/gene region, and arbitrarily specify a *K* rank truncation point so as to combine the *K* smallest *P* values as the summary statistics. Instead, ARTP takes into consideration the gene-based structure of biological pathways as well as the correlation between *P* values (which is estimated using an external panel of reference samples such as the 1000 Genomes Project), selects the optimal rank truncation point among a set of candidates and then adjusts the generated *P* value for multiple testing using a permutation procedure.

This gene-based pathway analysis first obtains a summary statistic for the association between each gene and the phenotype and then combines gene-level evidence using the ARTP method. A challenge for this approach is that it requires a multiple layer resampling procedure to calculate the significance of the pathway-level test statistic. In fact, a first layer of permutation is needed to generate the gene-level summary of association, a second layer is required to yield the *P* value associated with the pathway-level statistic for each truncation point and a third layer is necessary to assess the significance of the ARTP statistic after adjusting for multiple testing across different truncation points. Since this multi-level permutation procedure can become computationally intensive, the ARTP2 package implements an efficient algorithm using a single level of permutation iterations to achieve the goal of a multiple-level permutation procedure.

For the SNP selection process, we used a minor allele frequency (MAF) equal to or greater than 1% and a linkage disequilibrium *r*-squared lower than 0.9.

The number of candidate truncation points to inspect the top SNPs in a gene (or top genes in a pathway) was set at five, a truncation point being defined at every 20% of the top SNPs (or genes). In other words, considering the case of a gene (or pathway) represented by 100 SNPs (or genes), the five truncation points will be the following: 20, 40, 60, 80 and 100. The *P* values were estimated by 1,000,000 resampling steps. Since some degree of genomic over-dispersion is often observed under a polygenic model (even in the absence of population stratification and other technical artifacts) [16], the results were adjusted by the lambda inflation factor reported by each eligible GWAS. For each analysis, we reported the following information: (1) the pathway *P* value, with the number of SNPs and genes contributing to the pathway-level analysis; (2) the gene *P* value of each gene contributing to the pathway analysis, along with the number of SNPs contributing to the gene-level analysis; (3) the top gene and SNP, defined as the gene and the SNP with the lowest *P* value from the gene-level analysis and the original GWAS (or GWAS meta-analysis), respectively.

## Results

### GWAS

For each one of the three tumour types considered in this study, we found (and chose as the most informative data source) a meta-analysis of multiple GWASs:

1. *Breast cancer*. Data were available from the Discovery, Biology, and Risk of Inherited Variants in Breast Cancer (DRIVE, NCBI GaPdb accession number: pha004500) Consortium meta-analysis of 11 GWASs of breast cancer enrolling 15,748 cases affected with breast carcinoma and 18,084 controls of European ancestry [17]: the Australian Breast Cancer Family Study (ABCFS), the British Breast Cancer Study (BBCS), the Breast and Prostate Cancer Cohort Consortium (BPC3), the Breast Cancer Family Registry (BCFR), the Dutch Familial Bilateral Breast Cancer Study (DFBBCS), the German Consortium for Hereditary Breast and Ovarian Cancer (GC-HBOC), the Helsinki Breast Cancer (family) Study (HEBCS), the Mammary Carcinoma Risk Factor Investigation (MARIE), the Singapore and Sweden Breast Cancer Study (SASBAC), the Triple Negative Breast Cancer (TNBC) study and the UK2 GWAS. Illumina or Affymetrix platforms were utilized to genotype 9,331,393 SNPs. Separate data for patients with estrogen receptor negative tumour histology subtype were available from eight GWASs (cases, *n* = 4939; controls, *n* = 13,128).
2. *Prostate cancer*. Data were available from the Elucidating Loci Involved in Prostate Cancer Susceptibility (ELLIPSE, NCBI GaPdb accession number: pha004502.1) Consortium meta-analysis of six GWASs of prostate cancer enrolling 14,160 cases affected with prostate carcinoma and 12,724 controls of European ancestry [18]: the UK GWAS stage 1, the UK GWAS stage 2, the Cancer of the Prostate in Sweden 1 (CAPS1) study, the Cancer of the Prostate in Sweden 2 (CAPS2) study, the Breast and Prostate Cancer Cohort Consortium (BPC3) and the Prostate Cancer Genome-wide Association Study of Uncommon Susceptibility Loci (PEGASUS). Illumina or Affymetrix platforms were utilized to genotype 11,333,029 SNPs. Separate data for patients with aggressive tumour subtype (as defined by a Gleason score greater than 7, presence of distant metastasis, a prostate-specific antigen level greater than 100 ng/ml or death from prostate cancer) were available from all six GWASs (cases, *n* = 4450; controls, *n* = 12,724).
3. *Lung cancer*. Data were available from the Transdisciplinary Research In Cancer of the Lung (TRICL, NCBI GaPdb accession number: pha003883.1) Consortium meta-analysis of six GWASs of lung cancer enrolling 12,160 cases affected with lung carcinoma and 16,838 controls of European ancestry [19]: the Institute of Cancer Research (ICR) GWAS, the MD Anderson Cancer Center (MDACC) GWAS, the International Agency for Research on Cancer (IARC) GWAS, the National Cancer Institute (NCI) GWAS, the Samuel Lunenfeld Research Institute study (SLRI) and the Germany Lung Cancer study (GLC). Illumina platforms were utilized to genotype 318,094 to 543,697 SNPs. Separate data for squamous carcinoma (cases, *n* = 3422; controls, *n* = 16,015) and adenocarcinoma (cases, *n* = 3718; controls, *n* = 15,871) tumour histology subtypes were available from all six GWASs.

These data sources provided information on 15 out of 17 selected clock genes, as no SNPs were available for *CSNK1D* and *TIMELESS*. Overall, data on 181 SNPs were available (see Additional file 1: Table S1) and were utilized for ARTP-based pathway analysis, as described in the following section.

### ARTP-based gene and pathway analysis

As regards breast cancer (all cases), we found a highly significant association between circadian pathway variation and risk of developing this tumour (circadian pathway *P* value 1.9 × 10^–6^). This result was based on the data regarding 20 SNPs located in eight genes (Table 1). The top gene and SNP were *RORA* (eight SNPs, circadian gene *P* value 0.0003) and RORB rs1018584 (GWAS meta-analysis *P* value 0.0007), respectively.

**Table 1:** Results of ARTP-based gene analysis. Circadian genes statistically significantly associated with risk of cancer (all cases) are listed (ordered by increasing *P* value) along with the number of single nucleotide polymorphisms (SNPs) included in the analysis

Gene	Chromosome	No. SNPs	*P* value	Cancer
*RORA*	15	8	2.95E-04	Breast_all cases
*PER1*	17	2	3.34E-04	
*RORB*	9	5	6.49E-04	
*ARNTL*	11	1	0.002	
*CRY2*	11	1	0.002	
*CLOCK*	4	1	0.006	
*CRY1*	12	1	0.008	
*RORC*	1	1	0.010	
*ARNTL*	11	1	2.04E-04	Prostate_all cases
*RORA*	15	6	2.32E-04	
*NPAS2*	2	6	0.002	
*RORB*	9	1	0.005	
*NR1D1*	17	1	0.005	
*PER3*	1	1	0.009	
*CLOCK*	4	1	0.010	
*RORA*	15	27	2.00E-06	Lung_all cases
*RORB*	9	11	9.39E-05	
*ARNTL*	11	17	5.94E-04	
*NPAS2*	2	5	7.89E-04	
*CSNK1E*	22	4	7.97E-04	
*PER3*	1	3	0.002	
*PER2*	2	2	0.002	
*CLOCK*	4	3	0.003	
*CRY1*	12	2	0.007	
*CRY2*	11	1	0.008	
*NR1D1*	17	2	0.017	
*ARNTL2*	12	1	0.028	
*NR1D2*	3	1	0.046	

Upon subgroup analysis, the risk of estrogen receptor negative carcinoma was also associated with circadian pathway variation (circadian pathway *P* value: 2.4 × 10^–6^), the finding being based on 15 SNPs located in seven genes (Table 2). The top gene and SNP were *RORA* (seven SNPs, circadian gene *P* value 0.0002) and PER3 rs77404158 (GWAS meta-analysis *P* value 0.0003), respectively.

**Table 2:** Results of ARTP-based gene subgroup analysis. Circadian genes statistically significantly associated with risk of cancer subtypes (see text for more details) are listed (ordered by increasing *P* value) along with the number of single nucleotide polymorphisms (SNPs) included in the analysis

Gene	Chr	No. SNPs	*P* value	Cancer
*RORA*	15	7	2.11E-04	Breast_ER negative
*PER3*	1	1	3.14E-04	
*PER2*	2	3	0.001	
*CSNK1E*	22	1	0.002	
*ARNTL2*	12	1	0.004	
*NPAS2*	2	1	0.005	
*PER1*	17	1	0.009	
*RORA*	15	12	4.50E-06	Prostate_aggressive
*NPAS2*	2	5	9.75E-05	
*ARNTL*	11	2	1.90E-04	
*RORB*	9	3	9.25E-04	
*PER1*	17	1	0.002	
*PER3*	1	4	0.004	
*TIMELESS*	12	1	0.007	
*RORA*	15	55	1.50E-06	Lung_squamous
*RORB*	9	20	2.55E-05	
*NPAS2*	2	10	4.45E-05	
*ARNTL*	11	17	0.001	
*ARNTL2*	12	5	0.001	
*PER2*	2	3	0.003	
*NR1D2*	3	3	0.010	
*CLOCK*	4	2	0.012	
*CRY1*	12	1	0.023	
*TIMELESS*	12	3	0.033	
*RORC*	1	1	0.041	
*CSNK1E*	22	1	0.044	
*RORA*	15	45	2.00E-06	Lung_adenocarcinoma
*RORB*	9	17	8.50E-06	
*PER3*	1	4	5.03E-04	
*CLOCK*	4	2	0.001	
*ARNTL2*	12	3	0.001	
*ARNTL*	11	8	0.003	
*NR1D1*	17	4	0.005	
*NR1D2*	3	3	0.008	
*CSNK1E*	22	4	0.017	
*NPAS2*	2	2	0.021	
*PER1*	17	1	0.027	
*RORC*	1	3	0.029	
*CRY1*	12	1	0.037	

As for prostate cancer (all cases), there was a highly significant association between genetic variation of the circadian pathway and the susceptibility to this malignancy (circadian pathway *P* value 4.1 × 10^–6^). This result was based on the data regarding 17 SNPs located in seven genes (Table 1). The top gene and SNP were *ARNTL* (one SNP, circadian gene *P* value 0.0002) and ARNTL rs142435152 (GWAS meta-analysis *P* value 0.0002), respectively.

Subgroup analysis showed that the risk of aggressive prostate cancer was also associated with circadian pathway variation (circadian pathway *P* value 1.49 × 10^–6^), the finding being based on 28 SNPs located in seven genes (Table 2). The top gene and SNP were *RORA* (12 SNPs, circadian gene *P* value 4.49 × 10^–6^) and RORA rs17191414 (GWAS meta-analysis *P* value 0.000069), respectively.

As regards lung cancer (all cases), we found a highly significant association between genetic variation of the circadian pathway and the risk of developing this tumour (circadian pathway *P* value 6.9 × 10^–7^). This result was based on the data regarding 79 SNPs located in 13 genes (Table 1). The top gene and SNP were *RORA* (27 SNPs, circadian gene *P* value 2.0 × 10^–6^) and RORB rs77599950 (GWAS meta-analysis *P* value 0.0015), respectively.

Upon subgroup analysis, the risk of squamous carcinoma was associated with circadian pathway variation (circadian pathway *P* value 1.0 × 10^–6^), the finding being based on 121 SNPs located in 12 genes (Table 2). The top gene and SNP were *RORA* (55 SNPs, circadian gene *P* value 1.5 × 10^–6^) and RORB rs17684492 (GWAS meta-analysis *P* value 0.0006), respectively. Similarly, circadian pathway variation was also associated to the risk of adenocarcinoma (circadian pathway *P* value 9.9 × 10^–7^), the finding being based on 97 SNPs located in 13 genes (Table 2). The top gene and SNP were *RORA* (45 SNPs, circadian gene *P* value 2.0 × 10^–6^) and RORA rs73424095 (GWAS meta-analysis *P* value 0.000039), respectively.

**Fig. 2:**
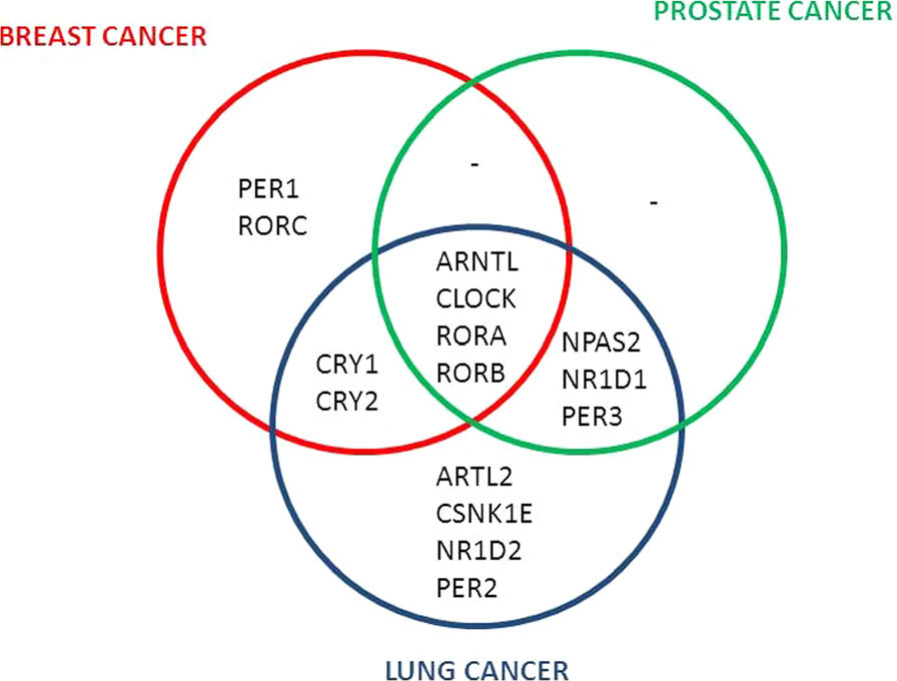
Venn diagram showing the genes selected by pathway analysis as statistically significantly associated with the risk of one, two or three types of cancer considered in this study

The genes statistically significantly linked to the risk of one, two or all three tumour types (breast, prostate and lung carcinoma) are illustrated in the Venn diagram of Fig. 2.

The details of ARTP-based gene analysis are reported in Additional file 2: Table S2 (primary analysis, all cases included) and Additional file 3: Table S3 (subgroup analysis by histological subtype).

## Discussion

In this study we found that germline genetic variation in the circadian pathway is associated with the risk of developing breast, prostate and lung carcinoma in a large cohort of cases (*n* = 42,068) and controls (*n* = 47,646). This association was also maintained in subgroup analyses for estrogen receptor negative breast cancer, aggressive prostate cancer and both squamous carcinoma and adenocarcinoma lung cancer. To the best of our knowledge, this is the first time that ARTP-based gene and pathway analysis has been applied to the relationship between circadian genes’ germline variation and cancer susceptibility. Thus far, molecular epidemiology studies have investigated only single variants of single circadian genes in relationship with some tumour types (such as breast, pancreatic and prostate carcinomas) [5, 20]. In particular, according to our recent systematic review and meta-analysis of the published literature on the subject [5], out of 687 SNPs (located in 14 circadian genes) only 10 SNPs located in five genes (NPAS2 rs10165970, rs895520, rs17024869 and rs7581886; CLOCK rs3749474 and rs11943456; RORA rs7164773 and rs10519097; RORB rs7867494; and PER3 rs1012477) resulted in being significantly associated with the predisposition to only one tumour type, that is, breast carcinoma. Moreover, none of the SNPs investigated in the three GWAS meta-analyses included in the present study reached statistical significance after adjustment for multiple testing [17–19]. In contrast, pathway analysis enabled us to link with high statistical significance (pathway *P* values always lower than 1 × 10^–5^) the circadian pathway variation to the susceptibility not only of breast cancer but also to that of other two most common malignancies such as prostate and lung carcinoma. This relationship was sustained by 15 statistically significant genes out of 17 genes investigated, with only *CSNK1D* and *TIMELESS* being excluded from the association (see Tables 1 and 2).

The implication of most circadian genes in all three tumour types (as well as all their subtypes) indicates that variation of this pathway could actually be involved in the predisposition to cancer in general, which still requires more investigation to be demonstrated in patients affected with malignancies other than those considered in this study. On the other hand, our results point out that the germline variation of some genes (*ARNTL*, *CLOCK*, *RORA* and *RORB*) is shared by all three tumour types, whereas the polymorphisms of other genes might be more specific to one or two malignancies (see the Venn diagram in Fig. 2). This finding suggests that some circadian genes might be more relevant than others in terms of cancer predisposition. In particular, it is noteworthy that all the above-mentioned four shared genes belong to the positive loop of the circadian pathway (that is, the stimulatory component of the biological clock circuit; see Fig. 1) and that *RORA* is the most significant gene associated with all tumour types (except for prostate carcinoma, where it ranks second) and subtypes (see Tables 1 and 2). However, the biological meaning of these observations requires dedicated studies to be elucidated. For instance, it is known that the *CLOCK* gene product activity can affect both estrogen [21] and androgen pathways [22], which is concordant with the relationship between circadian pathway perturbation and the risk of hormone-driven malignancies such as breast and prostate cancer, respectively; however, the association with lung carcinoma remains less intuitive and warrants further investigation on the cascade of molecular events underlying the link between the biological clock and this type of tumour.

Overall, our data underscore the fact that a biological relationship undetected by single polymorphisms can be unveiled by pathway analysis, confirming the power of this multi-SNP and multi-gene approach [8–10, 23].

In particular, our results support the pre-clinical evidence regarding the candidate role of the circadian pathway as a tumour suppressor circuit acting through the transcriptional control of (or the direct interaction with) key regulators of cell proliferation, apoptosis and DNA repair (and thus genomic stability) and metabolism, such as Ciclin-D1, c-Myc, Mdm2, p53, Gadd45-alpha, Atm, Chk1, Nampt and Sirt-1 [4, 24, 25], which are well known to play a pivotal role in carcinogenesis.

In a Mendelian randomization perspective (that is, using variation in genes of known function to examine the causal effect of a given environmental exposure/behaviour on disease, reasonably assuming that genes are not themselves associated with any confounding factors) [26, 27], our data also support the hypothesis that the disruption of the physiological internal clock — as in sleep deprivation, insomnia, work shifting and jet leg — might lead to an increased risk of cancer, as suggested by some classical epidemiology studies [28–31] and confuted by others [32–35], with most of them focusing on breast cancer.

Certainly, we cannot draw any definitive conclusion on this subject, as dedicated studies of fine mapping are needed to systematically investigate the relationship between germline variation of the circadian pathway molecular components and cancer risk. Moreover, functional experiments are required to fully dissect the actual link between circadian pathway polymorphisms and the molecular mechanisms underlying cancer development. Finally, a pathway-based polygenic risk score [36, 37] should be tested to translate genetic information into clinically valuable risk prediction. In fact, pathway analysis only provides evidence of association between a given biological circuit and the predisposition to a studied disease; it does not provide any clue to the magnitude of the risk linked to a specific (that is, individual) genetic signature. We hope that this study can represent a decisive step forward towards the personalization of cancer risk prediction, with potentially important implications in terms of screening programs [38].

## Conclusions

In conclusion, our results — based on pathway analysis of the largest series ever analysed in this research field — strengthen the already-existing (but discordant) clinical epidemiology data and genetic evidence (based on single polymorphisms) supporting the link between the genetic control of the circadian pathway and the development of cancer, which prompts further investigation in this promising area of cancer research.

## Additional files

Additional file 1: Table S1. Genes and corresponding single nucleotide polymorphisms (SNPs) investigated in the genome-wide studies included in our analysis. (DOCX 20 kb)

Additional file 2: Table S2. Adaptive rank truncated product (ARTP)-based analysis of single circadian genes: primary analysis (all cases included) by tumour type. (DOCX 15 kb)

Additional file 3: Table S3. Adaptive rank truncated product (ARTP)-based analysis of single circadian genes: subgroup analysis (by histological subtype) by tumour type. (DOCX 15 kb)

## Abbreviations

ARTP: Adaptive rank truncated product
GWAS: Genome-wide association study
SNP: Single nucleotide polymorphism

## References

[1] Partch CL, Green CB, Takahashi JS (2014). Molecular architecture of the mammalian circadian clock. Trends Cell Biol.

[2] Takahashi JS (2017). Transcriptional architecture of the mammalian circadian clock. Nat Rev Genet.

[3] Roenneberg T, Merrow M (2016). The circadian clock and human health. Curr Biol.

[4] Fu L, Lee CC (2003). The circadian clock: pacemaker and tumour suppressor. Nat Rev Cancer.

[5] Benna C, Helfrich-Forster C, Rajendran S, Monticelli H, Pilati P, Nitti D, Mocellin S (2017). Genetic variation of clock genes and cancer risk: a field synopsis and meta-analysis. Oncotarget.

[6] Visscher PM, Wray NR, Zhang Q, Sklar P, McCarthy MI, Brown MA, Yang J (2017). 10 years of GWAS discovery: biology, function, and translation. Am J Hum Genet.

[7] Civelek M, Lusis AJ (2014). Systems genetics approaches to understand complex traits. Nat Rev Genet.

[8] Wang K, Li M, Hakonarson H (2010). Analysing biological pathways in genome-wide association studies. Nat Rev Genet.

[9] Mooney MA, Nigg JT, McWeeney SK, Wilmot B (2014). Functional and genomic context in pathway analysis of GWAS data. Trends Genet.

[10] Ramanan VK, Shen L, Moore JH, Saykin AJ (2012). Pathway analysis of genomic data: concepts, methods, and prospects for future development. Trends Genet.

[11] Siegel RL, Miller KD, Jemal A (2017). Cancer statistics, 2017. CA Cancer J Clin.

[12] Little J, Higgins JP, Ioannidis JP, Moher D, Gagnon F, von Elm E, Khoury MJ, Cohen B, Davey-Smith G, Grimshaw J, Scheet P, Gwinn M, Williamson RE, Zou GY, Hutchings K, Johnson CY, Tait V, Wiens M, Golding J, van Duijn C, McLaughlin J, Paterson A, Wells G, Fortier I, Freedman M, Zecevic M, King R, Infante-Rivard C, Stewart A, Birkett N (2009). STrengthening the REporting of Genetic Association Studies (STREGA)—an extension of the STROBE statement. Genet Epidemiol.

[13] Subramanian A, Tamayo P, Mootha VK, Mukherjee S, Ebert BL, Gillette MA, Paulovich A, Pomeroy SL, Golub TR, Lander ES, Mesirov JP (2005). Gene set enrichment analysis: a knowledge-based approach for interpreting genome-wide expression profiles. Proc Natl Acad Sci U S A.

[14] Yu K, Li Q, Bergen AW, Pfeiffer RM, Rosenberg PS, Caporaso N, Kraft P, Chatterjee N (2009). Pathway analysis by adaptive combination of P-values. Genet Epidemiol.

[15] Zhang H, Wheeler W, Hyland PL, Yang Y, Shi J, Chatterjee N, Yu K (2016). A powerful procedure for pathway-based meta-analysis using summary statistics identifies 43 pathways associated with type II diabetes in European populations. PLoS Genet.

[16] Yang J, Weedon MN, Purcell S, Lettre G, Estrada K, Willer CJ, Smith AV, Ingelsson E, O'Connell JR, Mangino M, Mägi R, Madden PA, Heath AC, Nyholt DR, Martin NG, Montgomery GW, Frayling TM, Hirschhorn JN, McCarthy MI, Goddard ME, Visscher PM (2011). Genomic inflation factors under polygenic inheritance. Eur J Hum Genet.

[17] Fehringer G, Kraft P, Pharoah PD, Eeles RA, Chatterjee N, Schumacher FR, Schildkraut JM (2016). Cross-cancer genome-wide analysis of lung, ovary, breast, prostate, and colorectal cancer reveals novel pleiotropic associations. Cancer Res.

[18] Al Olama AA, Kote-Jarai Z, Berndt SI, Conti DV, Schumacher F, Han Y, Benlloch S, Hazelett DJ, Wang Z (2014). A meta-analysis of 87,040 individuals identifies 23 new susceptibility loci for prostate cancer. Nat Genet.

[19] Timofeeva MN, Hung RJ, Rafnar T, Christiani DC, Field JK, Bickeböller H, Risch A, McKay JD (2012). Influence of common genetic variation on lung cancer risk: meta-analysis of 14 900 cases and 29 485 controls. Hum Mol Genet.

[20] Kettner NM, Katchy CA, Fu L (2014). Circadian gene variants in cancer. Ann Med.

[21] Li S, Wang M, Ao X, Chang AK, Yang C, Zhao F, Bi H, Liu Y, Xiao L, Wu H (2013). CLOCK is a substrate of SUMO and sumoylation of CLOCK upregulates the transcriptional activity of estrogen receptor-a. Oncogene.

[22] Zhu Y, Zheng T, Stevens RG, Zhang Y, Boyle P (2006). Does “clock” matter in prostate cancer?. Cancer Epidemiol Biomarkers Prev.

[23] Kao PY, Leung KH, Chan LW, Yip SP, Yap MK (2017). Pathway analysis of complex diseases for GWAS, extending to consider rare variants, multi-omics and interactions. Biochim Biophys Acta.

[24] Sahar S, Sassone-Corsi P (2009). Metabolism and cancer: the circadian clock connection. Nat Rev Cancer.

[25] Shostak A (2017). Circadian clock, cell division, and cancer: from molecules to organism. Int J Mol Sci.

[26] Davey Smith G, Ebrahim S (2005). What can mendelian randomisation tell us about modifiable behavioural and environmental exposures?. BMJ.

[27] Didelez V, Sheehan N (2007). Mendelian randomization as an instrumental variable approach to causal inference. Stat Methods Med Res.

[28] Costa G (2015). Sleep deprivation due to shift work. Handb Clin Neurol.

[29] Haus EL, Smolensky MH (2013). Shift work and cancer risk: potential mechanistic roles of circadian disruption, light at night, and sleep deprivation. Sleep Med Rev.

[30] Thompson CL, Larkin EK, Patel S, Berger NA, Redline S, Li L (2011). Short duration of sleep increases risk of colorectal adenoma. Cancer.

[31] Davis S, Mirick DK (2006). Circadian disruption, shift work and the risk of cancer: a summary of the evidence and studies in Seattle. Cancer Causes Control.

[32] Markt SC, Grotta A, Nyren O, Adami HO, Mucci LA, Valdimarsdottir UA, Stattin P, Bellocco R, Lagerros YT (2015). Insufficient sleep and risk of prostate cancer in a large Swedish cohort. Sleep.

[33] Rod NH, Kumari M, Lange T, Kivimäki M, Shipley M, Ferrie J (2014). The joint effect of sleep duration and disturbed sleep on cause-specific mortality: results from the Whitehall II cohort study. PLoS One.

[34] Wu AH, Stanczyk FZ, Wang R, Koh WP, Yuan JM, Yu MC (2013). Sleep duration, spot urinary 6-sulfatoxymelatonin levels and risk of breast cancer among Chinese women in Singapore. Int J Cancer.

[35] Gallicchio L, Kalesan B (2009). Sleep duration and mortality: a systematic review and meta-analysis. J Sleep Res.

[36] Dudbridge F (2013). Power and predictive accuracy of polygenic risk scores. PLoS Genet.

[37] Lecarpentier J, Silvestri V, Kuchenbaecker KB, Barrowdale D, Dennis J, McGuffog L, Soucy P, Leslie G (2017). Prediction of breast and prostate cancer risks in male BRCA1 and BRCA2 mutation carriers using polygenic risk scores. J Clin Oncol.

[38] Pharoah PD, Antoniou A, Bobrow M, Zimmern RL, Easton DF, Ponder BA (2002). Polygenic susceptibility to breast cancer and implications for prevention. Nat Genet.

