# Supplementary material for: Circadian pathway genetic variation and cancer risk: evidence from genome-wide association studies
Source: BMC Med. 2018 Feb 19;16:20. doi: 10.1186/s12916-018-1010-1 (PMC5817863; doi:10.1186/s12916-018-1010-1)
Supplement: Supplementary file 1 — Genes and corresponding single nucleotide polymorphisms (SNPs) investigated in the genome-wide studies included in our analysis. (DOCX 20 kb) [file 12916_2018_1010_MOESM1_ESM.docx]

**Additional file 1: Table S1**

Genes and corresponding single nucleotide polymorphims (SNPs) investigated in the genome wide studies included in our analysis.

| **GENE** | **SNP** |
| --- | --- |
| ARNTL | rs10160685 |
| ARNTL | rs10741615 |
| ARNTL | rs11022734 |
| ARNTL | rs11022742 |
| ARNTL | rs11022752 |
| ARNTL | rs11022755 |
| ARNTL | rs11022765 |
| ARNTL | rs11022775 |
| ARNTL | rs117155878 |
| ARNTL | rs117629551 |
| ARNTL | rs142435152 |
| ARNTL | rs150663450 |
| ARNTL | rs1871835 |
| ARNTL | rs2279284 |
| ARNTL | rs2290036 |
| ARNTL | rs2290037 |
| ARNTL | rs34148132 |
| ARNTL | rs34834014 |
| ARNTL | rs56407311 |
| ARNTL | rs7104985 |
| ARNTL | rs7107287 |
| ARNTL | rs7123257 |
| ARNTL | rs7126225 |
| ARNTL | rs72867493 |
| ARNTL | rs72867496 |
| ARNTL | rs73423511 |
| ARNTL | rs7939846 |
| ARNTL | rs7949336 |
| ARNTL | rs900148 |
| ARNTL | rs998089 |
| ARNTL2 | rs4964057 |
| ARNTL2 | rs4964059 |
| ARNTL2 | rs76156375 |
| ARNTL2 | rs76706979 |
| ARNTL2 | rs78441302 |
| CLOCK | rs114621641 |
| CLOCK | rs116099505 |
| CLOCK | rs11932595 |
| CLOCK | rs17085763 |
| CLOCK | rs62303730 |
| CRY1 | rs114298876 |
| CRY1 | rs117524801 |
| CRY1 | rs139542230 |
| CRY1 | rs2374656 |
| CRY1 | rs77706154 |
| CRY2 | rs112302069 |
| CRY2 | rs113196716 |
| CRY2 | rs12281674 |
| CSNK1E | rs113908050 |
| CSNK1E | rs12484542 |
| CSNK1E | rs135750 |
| CSNK1E | rs135763 |
| NPAS2 | rs117207561 |
| NPAS2 | rs12619710 |
| NPAS2 | rs12622050 |
| NPAS2 | rs1542178 |
| NPAS2 | rs1542179 |
| NPAS2 | rs2278727 |
| NPAS2 | rs2278728 |
| NPAS2 | rs2305159 |
| NPAS2 | rs2305160 |
| NPAS2 | rs3739008 |
| NPAS2 | rs4851376 |
| NPAS2 | rs4851392 |
| NPAS2 | rs6707510 |
| NPAS2 | rs6719037 |
| NPAS2 | rs6747755 |
| NPAS2 | rs75405459 |
| NR1D1 | rs17616365 |
| NR1D1 | rs2071427 |
| NR1D1 | rs4795424 |
| NR1D1 | rs939347 |
| NR1D2 | rs114730050 |
| NR1D2 | rs4858564 |
| PER1 | rs2585408 |
| PER1 | rs3027191 |
| PER2 | rs116298301 |
| PER2 | rs145852943 |
| PER2 | rs146454363 |
| PER2 | rs77942338 |
| PER3 | rs12141043 |
| PER3 | rs2153733 |
| PER3 | rs76114366 |
| PER3 | rs77404158 |
| PER3 | rs79423628 |
| RORA | rs10459597 |
| RORA | rs10519080 |
| RORA | rs11631055 |
| RORA | rs11631432 |
| RORA | rs11634318 |
| RORA | rs11634359 |
| RORA | rs11634376 |
| RORA | rs116853819 |
| RORA | rs117333985 |
| RORA | rs117686196 |
| RORA | rs117823680 |
| RORA | rs117852591 |
| RORA | rs117957921 |
| RORA | rs11857128 |
| RORA | rs12324535 |
| RORA | rs12912031 |
| RORA | rs12913421 |
| RORA | rs12915127 |
| RORA | rs140805686 |
| RORA | rs141880306 |
| RORA | rs142885776 |
| RORA | rs143578611 |
| RORA | rs147344217 |
| RORA | rs147345547 |
| RORA | rs1482058 |
| RORA | rs149151277 |
| RORA | rs1523530 |
| RORA | rs16943453 |
| RORA | rs17191442 |
| RORA | rs17237563 |
| RORA | rs17270745 |
| RORA | rs17270752 |
| RORA | rs1869486 |
| RORA | rs1869488 |
| RORA | rs2247307 |
| RORA | rs2689352 |
| RORA | rs341403 |
| RORA | rs34735400 |
| RORA | rs34783737 |
| RORA | rs4267252 |
| RORA | rs4774360 |
| RORA | rs55754498 |
| RORA | rs59173136 |
| RORA | rs61740274 |
| RORA | rs66478217 |
| RORA | rs66507209 |
| RORA | rs66698157 |
| RORA | rs7166448 |
| RORA | rs7167514 |
| RORA | rs7183068 |
| RORA | rs7183916 |
| RORA | rs72739591 |
| RORA | rs72746677 |
| RORA | rs72754751 |
| RORA | rs7497885 |
| RORA | rs75503648 |
| RORA | rs75627031 |
| RORA | rs76125313 |
| RORA | rs76943880 |
| RORA | rs78949051 |
| RORA | rs79212114 |
| RORA | rs8033510 |
| RORA | rs962403 |
| RORA | rs9806633 |
| RORB | rs1018584 |
| RORB | rs10217594 |
| RORB | rs11143996 |
| RORB | rs11144030 |
| RORB | rs11144054 |
| RORB | rs113939372 |
| RORB | rs116991200 |
| RORB | rs117139176 |
| RORB | rs12378707 |
| RORB | rs12551356 |
| RORB | rs1327837 |
| RORB | rs1327839 |
| RORB | rs140669220 |
| RORB | rs1410225 |
| RORB | rs1410226 |
| RORB | rs144323436 |
| RORB | rs145083743 |
| RORB | rs17612183 |
| RORB | rs17684492 |
| RORB | rs17692795 |
| RORB | rs28672222 |
| RORB | rs3818559 |
| RORB | rs4376556 |
| RORB | rs57989758 |
| RORB | rs75797401 |
| RORB | rs76044749 |
| RORB | rs77599950 |
| RORB | rs7853479 |
| RORB | rs7873018 |
| RORC | rs143989282 |
| RORC | rs3828057 |
| RORC | rs4995918 |
