# Supplementary material for: Circadian pathway genetic variation and cancer risk: evidence from genome-wide association studies
Source: BMC Med. 2018 Feb 19;16:20. doi: 10.1186/s12916-018-1010-1 (PMC5817863; doi:10.1186/s12916-018-1010-1)
Supplement: Supplementary file 2 — Adaptive rank truncated product (ARTP)-based analysis of single circadian genes: primary analysis (all cases included) by tumour type. (DOCX 15 kb) [file 12916_2018_1010_MOESM2_ESM.docx]

**Additional file 2: Table S2**

Adaptive rank truncated product (ARTP) based analysis of single circadian genes: primary analysis (all cases included) by tumor type.

| **Gene** | **Chromosome** | **SNP** | **P-value** | **Cancer** |
| --- | --- | --- | --- | --- |
| RORA | 15 | 8 | 2.95E-04 | Breast_all |
| PER1 | 17 | 2 | 3.34E-04 | Breast_all |
| RORB | 9 | 5 | 6.49E-04 | Breast_all |
| ARNTL | 11 | 1 | 0.002 | Breast_all |
| CRY2 | 11 | 1 | 0.002 | Breast_all |
| CLOCK | 4 | 1 | 0.006 | Breast_all |
| CRY1 | 12 | 1 | 0.008 | Breast_all |
| RORC | 1 | 1 | 0.010 | Breast_all |
| ARNTL | 11 | 1 | 2.04E-04 | Prostate_all |
| RORA | 15 | 6 | 2.32E-04 | Prostate_all |
| NPAS2 | 2 | 6 | 0.002 | Prostate_all |
| RORB | 9 | 1 | 0.005 | Prostate_all |
| NR1D1 | 17 | 1 | 0.005 | Prostate_all |
| PER3 | 1 | 1 | 0.009 | Prostate_all |
| CLOCK | 4 | 1 | 0.010 | Prostate_all |
| RORA | 15 | 27 | 2.00E-06 | Lung_all |
| RORB | 9 | 11 | 9.39E-05 | Lung_all |
| ARNTL | 11 | 17 | 5.94E-04 | Lung_all |
| NPAS2 | 2 | 5 | 7.89E-04 | Lung_all |
| CSNK1E | 22 | 4 | 7.97E-04 | Lung_all |
| PER3 | 1 | 3 | 0.002 | Lung_all |
| PER2 | 2 | 2 | 0.002 | Lung_all |
| CLOCK | 4 | 3 | 0.003 | Lung_all |
| CRY1 | 12 | 2 | 0.007 | Lung_all |
| CRY2 | 11 | 1 | 0.008 | Lung_all |
| NR1D1 | 17 | 2 | 0.017 | Lung_all |
| ARNTL2 | 12 | 1 | 0.028 | Lung_all |
| NR1D2 | 3 | 1 | 0.046 | Lung_all |
