# Supplementary material for: Circadian pathway genetic variation and cancer risk: evidence from genome-wide association studies
Source: BMC Med. 2018 Feb 19;16:20. doi: 10.1186/s12916-018-1010-1 (PMC5817863; doi:10.1186/s12916-018-1010-1)
Supplement: Supplementary file 3 — Adaptive rank truncated product (ARTP)-based analysis of single circadian genes: subgroup analysis (by histological subtype) by tumour type. (DOCX 15 kb) [file 12916_2018_1010_MOESM3_ESM.docx]

**Additional file 3: Table S3**

Adaptive rank truncated product (ARTP) based analysis of single circadian genes: subgroup analysis (by histological subtype) by tumor type.

| **Gene** | **Chromosome** | **SNP** | **P-value** | **Cancer** |
| --- | --- | --- | --- | --- |
| RORA | 15 | 7 | 2.11E-04 | Breast_ER negative |
| PER3 | 1 | 1 | 3.14E-04 | Breast_ER negative |
| PER2 | 2 | 3 | 0.001 | Breast_ER negative |
| CSNK1E | 22 | 1 | 0.002 | Breast_ER negative |
| ARNTL2 | 12 | 1 | 0.004 | Breast_ER negative |
| NPAS2 | 2 | 1 | 0.005 | Breast_ER negative |
| PER1 | 17 | 1 | 0.009 | Breast_ER negative |
| RORA | 15 | 12 | 4.50E-06 | Prostate_aggressive |
| NPAS2 | 2 | 5 | 9.75E-05 | Prostate_aggressive |
| ARNTL | 11 | 2 | 1.90E-04 | Prostate_aggressive |
| RORB | 9 | 3 | 9.25E-04 | Prostate_aggressive |
| PER1 | 17 | 1 | 0.002 | Prostate_aggressive |
| PER3 | 1 | 4 | 0.004 | Prostate_aggressive |
| TIMELESS | 12 | 1 | 0.007 | Prostate_aggressive |
| RORA | 15 | 55 | 1.50E-06 | Lung_squamous |
| RORB | 9 | 20 | 2.55E-05 | Lung_squamous |
| NPAS2 | 2 | 10 | 4.45E-05 | Lung_squamous |
| ARNTL | 11 | 17 | 0.001 | Lung_squamous |
| ARNTL2 | 12 | 5 | 0.001 | Lung_squamous |
| PER2 | 2 | 3 | 0.003 | Lung_squamous |
| NR1D2 | 3 | 3 | 0.010 | Lung_squamous |
| CLOCK | 4 | 2 | 0.012 | Lung_squamous |
| CRY1 | 12 | 1 | 0.023 | Lung_squamous |
| TIMELESS | 12 | 3 | 0.033 | Lung_squamous |
| RORC | 1 | 1 | 0.041 | Lung_squamous |
| CSNK1E | 22 | 1 | 0.044 | Lung_squamous |
| RORA | 15 | 45 | 2.00E-06 | Lung_adenocarcinoma |
| RORB | 9 | 17 | 8.50E-06 | Lung_adenocarcinoma |
| PER3 | 1 | 4 | 5.03E-04 | Lung_adenocarcinoma |
| CLOCK | 4 | 2 | 0.001 | Lung_adenocarcinoma |
| ARNTL2 | 12 | 3 | 0.001 | Lung_adenocarcinoma |
| ARNTL | 11 | 8 | 0.003 | Lung_adenocarcinoma |
| NR1D1 | 17 | 4 | 0.005 | Lung_adenocarcinoma |
| NR1D2 | 3 | 3 | 0.008 | Lung_adenocarcinoma |
| CSNK1E | 22 | 4 | 0.017 | Lung_adenocarcinoma |
| NPAS2 | 2 | 2 | 0.021 | Lung_adenocarcinoma |
| PER1 | 17 | 1 | 0.027 | Lung_adenocarcinoma |
| RORC | 1 | 3 | 0.029 | Lung_adenocarcinoma |
| CRY1 | 12 | 1 | 0.037 | Lung_adenocarcinoma |
